# Supplementary material for: Shigella entry unveils a calcium/calpain-dependent mechanism for inhibiting sumoylation
Source: eLife. 2017 Dec 12;6:e27444. doi: 10.7554/eLife.27444 (PMC5745084; doi:10.7554/eLife.27444)
Supplement: Transparent reporting form [file elife-27444-transrepform.docx]

***eLife’s* transparent reporting form**

We encourage authors to provide detailed information *within their submission* to facilitate the interpretation and replication of experiments. If you have any questions, please contact us: editorial@elifesciences.org.

**Sample-size estimation**

•	You should state whether an appropriate sample size was computed when the study was being designed

•	You should state the statistical method of sample size computation and any required assumptions

•	If no explicit power analysis was used, you should describe how you decided what sample (replicate) size (number) to use

Please outline where this information can be found within the submission (e.g., page numbers or figure legends), or explain why this information doesn’t apply to your submission:

Our study is essentially based on *in vitro* approaches (cell lines), and there is no quantification associate to the unique experiment in infected newborn mice (only qualitative observation, Fig. 1H and I), thus sample size estimation information doesn’t apply to our submission.

**Replicates**

•	You should report how often each experiment was performed

•	You should include a definition of biological versus technical replication

•	The data obtained should be provided and sufficient information should be provided to indicate the number of independent biological and/or technical replicates

•	If you encountered any outliers, you should describe how these were handled

•	Criteria for exclusion/inclusion of data should be clearly stated

•	High-throughput sequence data should be uploaded before submission, with a private link for reviewers provided (these are available from both GEO and ArrayExpress)

Please outline where this information can be found within the submission (e.g., page numbers or figure legends), or explain why this information doesn’t apply to your submission:

- A clear statement of how often each experiment has been performed is indicated in the corresponding figure legend section.

- The number of independent experiments indicated in the figure legends section corresponds to independent biological replicates. In addition, technical replicates has been performed for each qPCR experiments (n=2) and gentamycin protection assays (n=3).

- Criteria for inclusion/exclusion: not applicable for our submission.

**Statistical reporting**

•	Statistical analysis methods should be described and justified

•	Raw data should be presented in figures whenever informative to do so (typically when N per group is less than 10)

•	For each experiment, you should identify the statistical tests used, exact values of N, definitions of center, methods of multiple test correction, and dispersion and precision measures (e.g., mean, median, SD, SEM, confidence intervals; and, for the major substantive results, a measure of effect size (e.g., Pearson's r, Cohen's d)

•	Report exact p-values wherever possible alongside the summary statistics and 95% confidence intervals. These should be reported for all key questions and not only when the p-value is less than 0.05.

- Statistical analysis method is described in the “material and methods section” (at page 8, line 183) and correspond to a two-tailed Student’s t-test and a two-tailed z-test (for Fig. 7B, D and F)

- For each figure presenting quantitative results (Fig. 1E, 1G, 5B, 5C, 5F, 5G, 6B, 6E, 6H, 7B, 7D, 7F, 1sup1B, 1sup1E, 4sup1), type of measures are indicated in the corresponding figure legend, and correspond to the mean + or – SD or SEM.

- p-values are indicated as exact values directly on the figure or as a range (no asterisk: p>0.05, *p<0.05, **p<0.01 and ***p<0.001) in each corresponding figure legend.

- Exact p-values are as follow:

Figure 1E: 30min: p=0.376, 60min: p=0.166, 90min: p=0.034, 120min: p=0.0074, 150min: p=0.0037.

Figure 1G: 30min: p=0.141, 60min: p=0.491, 90min: p=0.078, 120min: p=0.048, 150min: p=0.0011.

Figure 5B: siUbc9: p=0.008, siSAE2: p=0.030.

Figure 5C: siUbc9: p=0.0003, siSAE2: p=0.002.

Figure 5F: Ubc9 KO: p=0.007

Figure 5G: Ubc9 KO: p=0.001

Figure 6B: p=0.007

Figure 6E: p=0.00002

Figure 6H: Cdc42: p=0.003, RhoA: p=0.020, Rac1: p=0.006

Figure 7B: p=7.06x10^-14^

Figure 7D: p=0.0027

Figure 7F: p=0.86

Figure 1 supplement 1B: SUMO1 conjugates: p=0.0003, Free SUMO1: p=0.284

Please outline where this information can be found within the submission (e.g., page numbers or figure legends), or explain why this information doesn’t apply to your submission:

(For large datasets, or papers with a very large number of statistical tests, you may upload a single table file with tests, Ns, etc., with reference to page numbers in the manuscript.)

**Additional data files (“source data”)**

•	We encourage you to upload relevant additional data files, such as numerical data that are represented as a graph in a figure, or as a summary table

•	Where provided, these should be in the most useful format, and they can be uploaded as “Source data” files linked to a main figure or table

•	Include model definition files including the full list of parameters used

•	Include code used for data analysis (e.g., R, MatLab)

•	Avoid stating that data files are “available upon request”

Please indicate the figures or tables for which source data files have been provided:

Source data (e.g. numerical data that are represented as graphs in the paper) has been uploaded in a unique file (named source data E-life) and correspond to figures 1E, 1G, 5B, 5C, 5F, 5G, 6B, 6E, 6H, 7B, 7D, 7F and figure 1-supplement figure S1B, S1E, S4
